# Machine Learning Model Development and Evaluation for Non-contact Lower Limb Injury Risk Prediction in Elite Male Rugby Union: A Multi-team, Multi-season Analysis

**DOI:** 10.1186/s40798-026-01060-7

**Published:** 2026-08-26

**Authors:** Chris Leckey, Nicol van Dyk, Jakim Berndsen, Cailbhe Doherty, Aonghus Lawlor, Eamonn Delahunt

**Affiliations:** 1 School of Public Health, Physiotherapy & Sports Science, University College Dublin, Dublin, Ireland; 2 High Performance Unit, Irish Rugby Football Union, Dublin, Ireland; 3 Section Sports Medicine, Faculty of Health Sciences, University of Pretoria, Pretoria, South Africa; 4 Insight Centre for Data Analytics, University College Dublin, Dublin, Ireland; 5 Institute for Sport and Health, University College Dublin, Dublin, Ireland

## Abstract

**Background:**

The prevalence, incidence rate and burden of injuries are high in rugby union. Despite the growing application of machine learning for injury risk analysis in sport, its use in rugby union has been primarily confined to single-team and single-season study cohorts. The aim of this study was to evaluate the performance of machine learning-based injury risk prediction models in elite male rugby union and to interpret their potential applicability in professional sporting environments.

**Methods:**

Multi-season data (2021/22–2023/24) relating to 129 professional male rugby union players from the four Irish provincial teams (Connacht Rugby, Leinster Rugby, Munster Rugby and Ulster Rugby), along with the Ireland men’s national team, were included in the analyses. External workload data from Global Navigation Satellite System (GNSS) units, self-perceived wellness scores, and musculoskeletal screening measures were modelled using logistic regression, Support Vector Machine (SVM), Random Forest, Extreme Gradient Boosting (XGBoost), and Categorical Boosting (CatBoost). ‘Injury’ was defined as a non-contact, soft-tissue injury to the lower limb, with incidents logged by team medical staff in a centralised athlete management system.

**Results:**

CatBoost achieved the highest values of area under the receiver operating characteristic curve (ROC AUC = 0.66), area under the precision-recall curve (AUPRC = 0.008, prevalence = 0.0039), and precision (0.013).

**Conclusion:**

Predictive performance across the models was poor, with low precision scores highlighting each model’s inability to effectively identify impending injuries. The resulting high false-positive rate (1 correct prediction for every 77 false predictions) underscores the limited applicability of the tested machine learning approaches within the modelling framework analysed in this study.

**Supplementary Information:**

The online version contains supplementary material available at 10.1186/s40798-026-01060-7.

## Key points

- Gradient boosted decision tree models Categorical Boosting (CatBoost) and XGBoost (Extreme Gradient Boosting) provided the best performance of the tested models, consistent with existing literature.
- Non-modifiable risk factors, including age, injury history, and session type (i.e. ‘match’ or ‘training’) accounted for four of the top five most important features based on SHapley Additive exPlanations (SHAP) analysis.
- Despite exposure to diverse, granular datasets, relating to multiple teams across multiple seasons, the tested machine learning models produced “poor” predictive ability.

**Supplementary Information:**

The online version contains supplementary material available at 10.1186/s40798-026-01060-7.

## Introduction

The game of rugby union is characterised by high-intensity actions (e.g., accelerations, decelerations, sprints, and collisions), interspersed with periods of lower intensity actions (e.g., jogging, walking) and recovery [1]. Rugby union is the most widely played contact sport globally [2], involving a unique combination of frequent collisions (e.g. tackles, scrums, rucks and mauls) alongside intermittent running demands [3]. Players cover an average of 5.6 kms per game [4], although this varies by position (forwards = 5.3, backs = 5.9 [4]) and competition level (domestic = 5.8, international = 5.4 [3]). Injuries within rugby union are common, with the incidence rate in the professional game (91 per 1000 h) [5] exceeding those reported in other elite-level field-based sports such as soccer (36 per 1000 h) [6] and Gaelic Football (56 per 1000 h) [7], thus reflecting the distinct physical demands of the sport. Rugby injuries are most commonly sustained to the lower limb [8], with high soft-tissue injury incidence rates reported [9]. A reported match-related hamstring injury incidence rate of 5.6 per 1000 player hours [10]—comparable to those reported in soccer (match incidence of 4.9 per 1000 player hours) [11]—indicates the considerable high-speed running demands associated with rugby union. Injuries in rugby union have a negative association with on-field performance. Research has demonstrated that increased injury burden (the product of injury incidence rate and injury severity) led to poorer league performance for teams participating in the professional English Premiership [12].

The protection of player health and the prevention of sport-related injuries are key goals of both the International Olympic Committee (IOC) and World Rugby [13, 14]. Both organisations recognise effective injury and illness surveillance as fundamental to the protection of athlete health, and promote data sharing between research groups. The IOC consensus statement encourages the use of data analytics, referring to the theories, technologies, tools and processes that enable in-depth understanding and discovery of actionable insight into data [15], to advance the management of sports injuries [13]. The application of data analytics in professional sport has been facilitated by the proliferation of electronic performance and tracking systems, leading sporting organisations and researchers to seek data-driven solutions to a variety of problems.

Machine learning (ML), a subcategory of Artificial Intelligence (AI) concerning the investigation of data to uncover underlying patterns [16], has been used within rugby union to support in-game decision-making [17], investigate performance metrics associated with victory [18], and identify tackle events associated with concussion [19]. The need to account for the non-linearity and complexity of injury aetiology has led to the application of ML to injury risk prediction in sport [20]. The ability of ML to handle high-dimensional datasets, such as those gathered by electronic performance and tracking systems, allows for the development of more complex models, compared to traditional statistical analyses [21]. Despite the growth in ML applications for injury risk prediction in sport, relevant research in rugby union remains sparse [22], hampered by single- team or single-season analyses, which limit the generalisability of the generated models [23, 24]. Although existing ML approaches to sports injury prediction have reported acceptable discrimination, their real-world utility is undermined by heterogenous definitions of injury and broad prediction windows [22].

Accordingly, this research aims to address these gaps by adopting a consensus-based definition of injury and synthesising longitudinal data from multiple professional rugby union teams across a three-season study period. We evaluate whether ML-based injury risk models, selected for their capacity to capture complex relationships within high-dimensional data, demonstrate predictive performance capable of supporting decision-making in elite male rugby union. In this context, performance evaluation extends beyond model discrimination to encompass precision (i.e., correctly identifying true injury cases), explainability (e.g., feature importance), and the consequences of false-positive predictions (i.e., injury overprediction) for practitioner behaviour.

## Methodology

### Study Design

This was a prospective cohort study design. A completed TRIPOD + AI (Transparent Reporting of a multivariable prediction model for Individual Prognosis or Diagnosis) checklist is included in Supplementary File S2. All data analysed were collected as part of the regular provision of medical and athletic performance services by each of the four professional provincial male rugby union teams (Connacht Rugby, Leinster Rugby, Munster Rugby and Ulster Rugby), and the Ireland men’s national team, under the aegis of the Irish Rugby Football Union (IRFU). The IRFU made the following datasets available for analysis: (i) external workload data gathered using wearable Global Navigation Satellite System (GNSS) units, worn during training sessions and matches (including United Rugby Championship, Champions Cup, Challenge Cup, Six Nations Championship, Rugby World Cup 2023, Autumn Nations Series, and Summer Tour fixtures), (ii) players’ self-perceived wellness measures reported on training and match days, (iii) pre- and in-season musculoskeletal test results, and (iv) athlete injury and illness records via the IRFU’s centralised athlete management system. These datasets related to the three consecutive seasons 2021/22, 2022/23 and 2023/24, including pre-seasons. Patients and/or the public were not involved in the design, conduct, reporting or dissemination plans of our research.

### Study Sample

At any time during the three-season study period, a total of 281 players registered competitive match exposure for any of the five professional teams listed above. To ensure longitudinal completeness across data domains, inclusion was restricted to players with recorded training or match GNSS, wellness, and musculoskeletal testing data across all three seasons. This approach prioritised data integrity by retaining only those who remained within the player cohort for the three-season study period.

### Definition of Injury

For the purposes of this study, ‘injury’ was defined as any non-contact, lower limb soft-tissue injury that resulted in time loss. A non-contact injury is an injury that occurs without direct contact to, or collision with, another individual [25]. A time-loss injury is an injury whereby the player was unable to take part in the following training session or competitive match, as per the consensus statement of Fuller [26]. The injuries included within the definition of ‘lower limb, soft-tissue’ were those sustained to the muscles, ligaments, or tendons of the foot, ankle, lower leg, knee, thigh and hip/groin, in accordance with the IOC consensus statement [13].

### Injury Data Collection

Medical records relating to the in-scope players were extracted from the IRFU’s athlete management system, in which all provincial injury incidents are centrally collated. Injury incidents were logged by provincial medical staff on a per-event basis during regular provision of medical service delivery. To determine those referring to lower limb soft-tissue injuries, analysis of Orchard Sports Injury and Illness Classification System (OSIICS) codes and medical notes from clinicians was conducted by CL, ED, and NvD [27, 28]. For cases where the nature of the injury was unclear from the clinical notes, CL analysed match footage to determine whether the inciting event was a contact or non-contact incident. Instances in which the nature of the event was either contact related, or unclear, were excluded from the analysis.

### Independent Variables

External workload data were collected using STATSports (Newry, Northern Ireland) Apex 10 Hz GNSS units, for all on-field training and match sessions, for each team in the study. Prior research has reported the “excellent” inter-unit reliability of these devices [29].

Self-perceived wellness data were collected from players during the course of each season on training days. These data were collected using a mobile application with which each player recorded Likert scores ranging from 0 to 10 for perceived measures of sleep quality, appetite, fatigue, stress, muscle tightness, and soreness.

Musculoskeletal range of motion and strength assessments were administered by team medical staff throughout the course of the study period as routine screening tests, with frequency varying according to team schedules and clinical indication. These tests comprised (i) ankle dorsiflexion (via a knee-to-wall test [30], measured in degrees), (ii) sit and reach (measured using a sit and reach box [31], reported in centimetres), and (iii) groin squeeze strength (measured using an adductor squeeze test at 45° hip flexion [32], reported in millimetres of mercury using a sphygmomanometer), with maximal values reported for each.

The independent variables retained for model development are listed in supplementary table S1.

The initial GNSS dataset consisted of 234 numeric variables relating to multiple dimensions of athlete workload and exposure, including total distances covered, maximal speed, metabolic load, acceleration-deceleration demands, and contact-related metrics. The proportion of missing data was assessed for each feature, and in the absence of a universal threshold for missingness [33], a conservative threshold of 20% was chosen to mitigate bias while retaining a substantial feature set of 89 variables.

### Collinearity Checks

All independent variables were assessed for multicollinearity using Pearson Correlation coefficients, with a threshold of 0.7 applied [34]. GNSS feature pairs exceeding this threshold were evaluated, with the decision as to which to retain based on theoretical relevance to lower-limb, soft-tissue injury risk (i.e. high-speed running exposure [35]), and to preserve a representative range of metrics capable of capturing rugby-specific workload dynamics (i.e., collision load and high metabolic load distance [3]). Additionally, the Variance Inflation Factor (VIF) for each of the remaining features was determined, with VIF exceeding a value of five removed from the analyses [36].

### Outlier Analysis

Outliers in the GNSS data were defined as values above three standard deviations from the average match demands across a player’s positional cohort. This threshold was applied as a pragmatic means of identifying implausibly high values (i.e., those resulting from sensor malfunction), while retaining rare but valid high-load observations, such as those games extended into extra time. This procedure resulted in the removal of 2% of the GNSS records. Information relating to the GNSS features retained following data preprocessing is included in Table 1.

**Table 1:** Summary of GNSS features retained following data preprocessing

GNSS measure name	Description
Acceleration impulse	Acceleration intensity per minute calculated using cumulative magnitude divided by number of occurrences
Accelerations (Zone 5–Zone 6)	Accelerations between 4 and 10 m/s^2^ with a minimum duration of 0.5 s
Average metabolic power	Average power output in W/kg of bodyweight per second during the session
Collision load	Calculation of stress and toughness of collisions based on magnitude and duration of each collision
Decelerations (Zone 5–Zone 6)	Decelerations between 4 and 10 m/s^2^ with minimum duration of 0.5 s
Distance covered (Zone 6 relative)	Distance covered in relative speed zone 6 (85–100% of individualised maximal velocity)
Dynamic stress load	Weighted total of accelerometry above 2G based on convex curved G-force ratings
Fatigue index	Speed Intensity score (the measure of total exertion based on time spent in each of the speed zones) divided by Dynamic Stress Load
High metabolic load distance	Distance covered accelerating/decelerating above 2 m/s^2^ magnitude or above 5.5 m/s
High-speed running per minute (relative)	Distance covered travelling above relative speed zone 5 (70–100% of individualised maximal velocity) threshold per minute
Lower speed loading	All accelerometer loading recorded while travelling at speeds lower than 2 m/s
Max speed	Maximum speed attained per day
Sprint distance	Distance covered at a speed of ≥ 5.5 m/s (minimum duration of a sprint is 1 s)
Total time	Total duration (minutes) of training sessions and/or matches completed

*GNSS* Global Navigation Satellite System, *m* Metres, *s* Seconds, *W* Watts, *kg* Kilograms, *G* Gravitational force

Wellness data were collected using a mobile application that limited responses between 0 and 10, ensuring that all entries fell within the valid range. Outlier removal was not applied as extreme responses were considered clinically relevant rather than noise.

For musculoskeletal testing records, the intra-player variances for each of the assessments were calculated (per player, per test type), with players exhibiting the highest variance flagged for manual review. These cases were reviewed by CL, ED, and NVD, with data entry errors identified as those exceeding the limits of the measurement instruments. Implausible measurements (i.e., groin squeeze erroneously logged as 3000 in place of 300, inconsistent with a player’s typical range), were manually corrected. Manual corrections were applied to 0.1% of test scores.

### Data Imputation

The pattern of missingness was assessed using the Amelia package in R (Version 4.3.3; R Foundation for Statistical Computing, Vienna, Austria). Visual inspection of the resulting missingness maps suggested a Missing at Random (MAR) pattern. Given that the MAR assumption cannot be formally tested [37], it was considered plausible for GNSS missingness to be assumed MAR, and the missing GNSS data to be imputed via Multiple Imputation by Chained Equations (MICE) using Predictive Mean Matching (PMM) via the *mice* package in R (imputations = 5, maximum iterations = 10). Only GNSS-derived workload features were used in the MICE imputation model.

Based on its suitability to the imputation of Likert scale data [38], K-Nearest Neighbours (KNN) was applied for imputation of the wellness data, using the scikit-learn package in Python (*k* = 5).

To account for instances whereby one or more of the musculoskeletal tests were missing on a given day, a method of last observation carried forward (LOCF) was applied under the assumption that the athlete’s strength or range of motion had not changed since the point of last assessment [39].

### Data Integration

The procedure outlined above resulted in fourteen GNSS metrics, six wellness scores, and three musculoskeletal tests being retained for analysis. Non-modifiable risk factors including body mass, height, age, playing position, were also included. In addition, features capturing each player’s injury history, including a rolling count of in-scope injuries sustained during the study and days since the most recent in-scope injury. Features relating to medical illness and concussion histories were also incorporated (see supplementary Table S1).

To construct the combined dataset, a complete longitudinal data panel was created, representing one row for each player, for each day of the study period. GNSS-derived data were outer joined to this panel, with days for which no workload was recorded set to zero for the external workload features, representing scheduled rest days. Wellness data were subsequently outer joined. Days on which no wellness was recorded were imputed using the mean of the five most recent records for that player, reflecting a short-term rolling average, as imputing zeros in this instance would artificially indicate poor wellness states (i.e., low sleep quality or high stress). Finally, the musculoskeletal testing data were outer joined to the composite dataset, with test values filled forward until the next recorded change in strength or range of motion.

### Feature Engineering

To capture temporal patterns within this data structure, exponentially weighted moving averages (EWMA) for the GNSS, wellness, and musculoskeletal data were calculated for each day in the three-season composite data. Features were generated for 3-, 7-, 14-, 21-, and 28-day windows (smoothing factors of 0.50, 0.25, 0.133, 0.091, and 0.069 respectively) to reflect short- and long-term fluctuations. The 7- to 28-day windows were selected to align with the windows underpinning the Acute:Chronic Workload Ratio (ACWR) framework [40], while the 3-day span was included to capture within-week training load variability. Additionally, the cumulative 7-day loads for these features were calculated, as well as the percentage change between these 7-day cumulative loads, versus the prior 7-day period. The models were not exposed to any data collected on the day of injury to preserve appropriate temporal sequencing between predictors and outcome.

### Data Scaling

To appreciate the anthropometric heterogeneity of rugby players, a dual approach was adopted for data scaling. Population relevant measures such as age, cumulative injury history, body mass, and height were scaled relative to all players in the study, using min–max scaling. All external GNSS workload, self-perceived wellness, and musculoskeletal testing measures were min–max scaled relative to each player to reflect the magnitude of fluctuations, relevant to each individual’s normal values. To prevent information leakage, feature scaling was applied in a fold-safe manner, whereby scaling parameters were estimated on training data only and subsequently applied to the validation and testing data.

### Choice of Classification Models

Injury risk prediction is most commonly approached as a classification problem, whereby models are developed to assign records to a particular class (i.e. ‘injury’ or ‘no injury’), with 92% of related research applying this framework [22]. The application of tree-based, ensemble methods has yielded strong performance when applied to injury risk prediction in sport, with Random Forest and Extreme Gradient Boosting (XGBoost) both commonly utilised classification methods [22]. Recently, other state-of-the-art gradient boosting methods, such as Categorical Boosting (CatBoost), have also been applied [41]. Logistic regression and Support Vector Machines (SVM) were reported as providing the best predictive performance of the non-tree-based ensemble methods in the classification domain [22]. As such, logistic regression and SVM were selected as comparative linear and kernel-based benchmarks against the tree-based, ensemble ML approaches. Accordingly, models were created for logistic regression, SVM, Random Forest, XGBoost, and CatBoost. To mitigate the effects of class imbalance, cost-sensitive learning was applied to each algorithm via class-weighted loss functions, assigning greater weight to the minority class (i.e. ‘injury’) observations during model training.

**Fig. 1:**
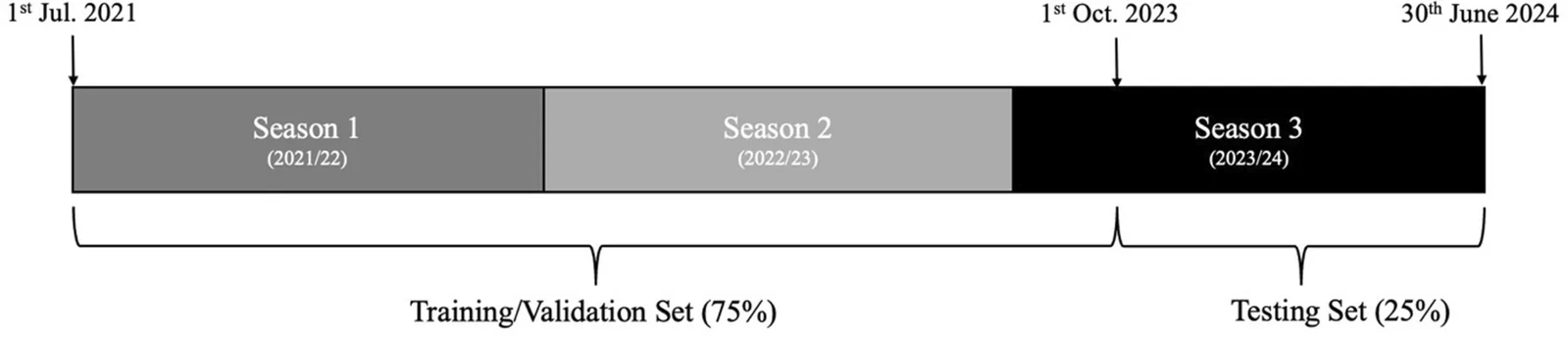
Study timeline

To prevent information leakage, a temporal train/test split was applied. The 1st October 2023 was selected as the data partition boundary as it represented a natural break in the dataset, preceding the first competitive matches of the 2023/24 season (21st October 2023). All data captured before 1st October 2023 was used to train the model, all data after this date comprised the testing set, which yielded a 75%/25% train/test split (Fig. 1), consistent with commonly used data partitioning strategies in ML, where no single, optimal ratio exists. The data were modelled as independent observations, with temporal structure captured through engineered features, rather than explicit time-series modelling.

### Cross-validation and Hyperparameter Optimisation

To assess the generalisability of each model, a process of stratified three-fold nested cross-validation was applied, using the same number of folds for the inner and outer loops. The outer loop was repeatedly partitioned into folds, with one fold retained as a hold-out validation set, and the remaining folds used for model training. Stratified cross-validation was applied to retain the class balance structure of the data. Fold-specific scaling was applied to the validation data, with parameters learned from the training fold only. Within each training partition, an inner cross-validation loop was used to perform hyperparameter optimisation using the Optuna framework (trials = 30) [42], with model performance assessed on the held-out inner validation fold. Following each outer loop evaluation, model hyperparameters were aggregated (mean for continuous or integer parameters, mode for categorical parameters) to produce a set of hyperparameters that would be applied to the final model training on the full training set. This approach reduces optimistic bias arising from hyperparameter tuning [43]. Model hyperparameters and F1-score optimised decision thresholds are included in the supplementary materials S1.

### Model Evaluation and Explainability

Models were developed to predict whether a non-contact, lower limb soft-tissue injury occurred in the subsequent training session or match (i.e., a 1-day prediction window aligned to exposure days). Predictions were not generated for rest days, as no injury events were logged on days without training or match exposure therefore no opportunity for injury exists. To support this design, the feature space was restricted to the engineered, temporal features for EWMA, 7-day load, and weekly percentage change variables for the GNSS, wellness and musculoskeletal testing features listed in Table S1. The inclusion of daily values would otherwise introduce systematic bias by pairing valid, high-load training or match sessions, with the inherently null injury risk associated with a subsequent rest day. The direction of this structural bias would therefore be toward the underestimation of injury risk following higher load sessions. Additionally, the removal of predictions for rest days prevents optimistic model performance arising from correct predictions of ‘no injury’ on rest days.

Model explainability was assessed post hoc using SHapley Additive exPlanations (SHAP) to visualise the feature importance for the highest performing model, when applied to the composite, held-out test set [44]. Statistical performance was assessed based upon each model’s area under the receiver operating characteristic curve (ROC AUC), area under the Precision-Recall Curve (AUPRC), precision, recall (sensitivity), and F1-Score. Equations for, and explanations of each of these metrics are provided in the supplementary material, in addition to the Cumulative Accuracy Profile (CAP) curve for the model with the highest statistical performance [45].

## Results

### Participants and Injuries

**Fig. 2:**
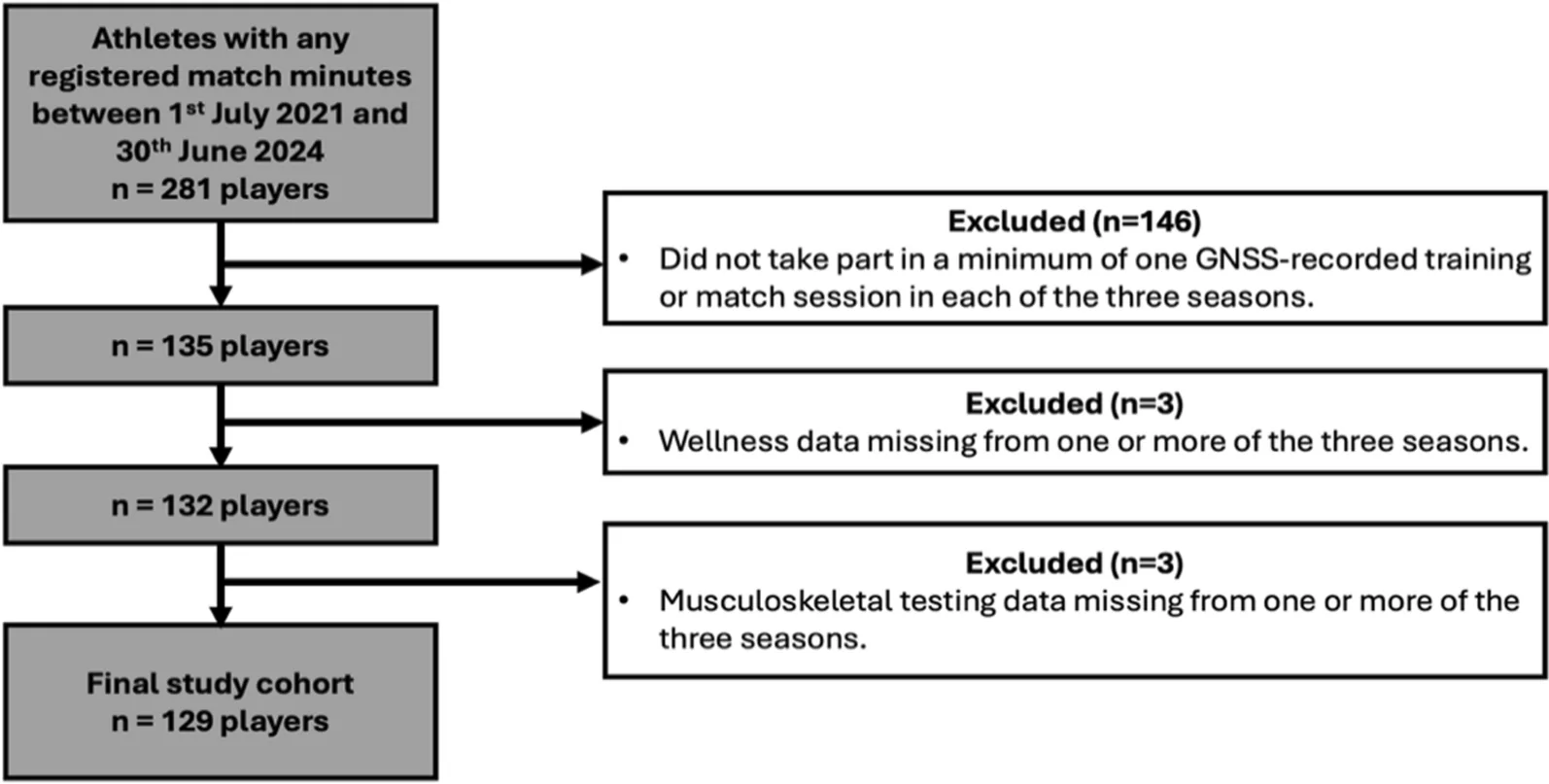
Player selection flowchart. *GNSS* Global Navigation Satellite System

The player inclusion criteria, as outlined in Fig. 2, were applied leaving a study cohort of 129 professional rugby players from the four provincial teams (see Table 2 for cohort demographic data). The median number of weekly training sessions per athlete was three (IQR: 2–4), while the median number of games per player each season was 16 (IQR: 9–21). Overall, GNSS data were available for 47,547 player training sessions and 5,773 match exposures. Wellness data were logged with a median of three weekly records per player (IQR: 2–3), resulting in 39,034 days of logged self-perceived wellness data. The median number of monthly musculoskeletal assessments per athlete was seven (IQR: 3–11). The integrated dataset totalled 141,384 records (1096 days of data for 129 players).

**Table 2:** Demographic data of all players (*N* = 129)

Age (years)	24.8 ± 3.8
Height (m)	1.87 ± 0.07
Body mass (kg)	104.3 ± 12.4
Position	
Forward	73 (57)
Front row	35 (27)
Second row	16 (12)
Back row	22 (17)
Back	56 (43)
Half backs	22 (17)
Centres	12 (9)
Back three	22 (17)

Data reported as mean ± standard deviation or *N* (%)

*m* Metres, *kg* Kilograms

The application of the previously stated criteria for lower limb, non-contact, soft tissue injuries led to a reduction in the number of time-loss injuries for analysis from 1,047 to 261. A summary of the body areas to which injuries were reported is provided in Table 3. The temporal train/test split allocated 207 (79%) injuries for model training and cross-validation, with the remaining 54 (21%) reserved for testing.

**Table 3:** Injuries by OSIICS body area (*N* = 261)

Thigh	111 (43%)
Lower leg	79 (30%)
Hip and groin	23 (9%)
Ankle	22 (8%)
Foot	15 (6%)
Knee	11 (4%)
**Total**	**261**

Data reported as *N* (%)

The median number of non-contact lower limb soft-tissue injuries sustained by each player within the cohort during the three-season study period was two (IQR: 1–3). The maximum number of injuries sustained by a single player was nine (*n* = 2). Twenty-six players (20%) sustained no non-contact lower limb soft-tissue injuries across the study period. No single player contributed more than 4% of the total injury events. When normalised to positional cohort size, ‘Back Three’ players had the highest injury rate per player of 2.64 (*n* = 58), followed by ‘Half Backs’ (2.45 injuries per player, *n* = 54), and ‘Centres’ (1.92 injuries per player, *n* = 23). For the forward positions, the ‘Front Row’ experienced 1.80 injuries per player (*n* = 63), with the ‘Back Row’ and ‘Second Row’ exhibiting rates of 1.77 and 1.50 injuries per player respectively (*n* = 39, 24). The median time loss duration of the in-scope injuries was 13 (IQR: 7–28) days, with the most severe injury resulting in a time loss of 160 days. Injury incidence rates were calculated using exposure hours derived from GNSS-based session durations, resulting in rates of 12.6 per 1000 h and 2.4 per 1000 h for matches and training sessions, respectively.

### Predictive Model Performance

Table 4 summarises the statistical performance of the five predictive modelling approaches during cross-validation and testing phases. ROC AUC, the most commonly reported evaluation metric in existing literature (71% of studies) [22], is defined on a scale from 0.5 (performance equivalent to random chance) to 1 (perfect discrimination). AUPRC, which is often reported in the presence of class imbalance [46], is more sensitive to the proportion of positive-class instances in the data (prevalence). As such, AUPRC should be assessed relative to prevalence, rather than fixed thresholds. These measures, alongside precision which reflects a model’s ability to correctly identify injury instances, are bolded to improve readability.

**Table 4:** Statistical evaluation of ML models during cross-validation and testing

Model	Stratified threefold nested cross-validation	Hold-out test set					
	ROC AUC	ROC AUC	F1-score	Precision	Recall	AUPRC	Prevalence
Logistic regression	0.49 ± 0.02	**0.58**	0.011	**0.005**	0.407	**0.005**	0.0039
SVM	0.59 ± 0.02	**0.52**	0.008	**0.005**	0.019	**0.005**	
Random forest	0.62 ± 0.02	**0.58**	0.01	**0.005**	0.352	**0.005**	
XGBoost	0.58 ± 0.01	**0.61**	0.021	**0.011**	0.167	**0.007**	
CatBoost	0.61 ± 0.02	**0.66**	0.024	**0.013**	0.148	**0.008**	

*ROC* Receiver operating characteristic, *AUC* Area under the curve, *AUPRC* Area under the precision-recall curve, *SVM* Support Vector Machine, *XGBoost* Extreme Gradient Boosting, *CatBoost* Categorical Boosting

Overall, the models yielded “poor” predictive ability, defined by Sturdivant and Hosmer [47] as those attaining ROC AUC between 0.5 and 0.7. Of the methods tested, CatBoost provided the highest statistical performance when applied to the unseen testing data, achieving values of ROC AUC (0.66), AUPRC (0.008, prevalence = 0.0039), and precision (0.013). XGBoost was the only other model to surpass a test ROC AUC of 0.6, achieving 0.61, with AUPRC and precision values of 0.007 and 0.011, respectively, comparable to those of the CatBoost model. Kernel-based SVM achieved the lowest ROC AUC during testing, while logistic regression was found to match the performance of Random Forest when applied to the test set yet failed to outperform chance during cross-validation. This fluctuation between cross-validation and test ROC AUC of 0.09, the highest of the models evaluated, likely reflects high variance in performance estimation under severe class imbalance and low signal-to-noise conditions.

### Feature Importance

**Fig. 3:**
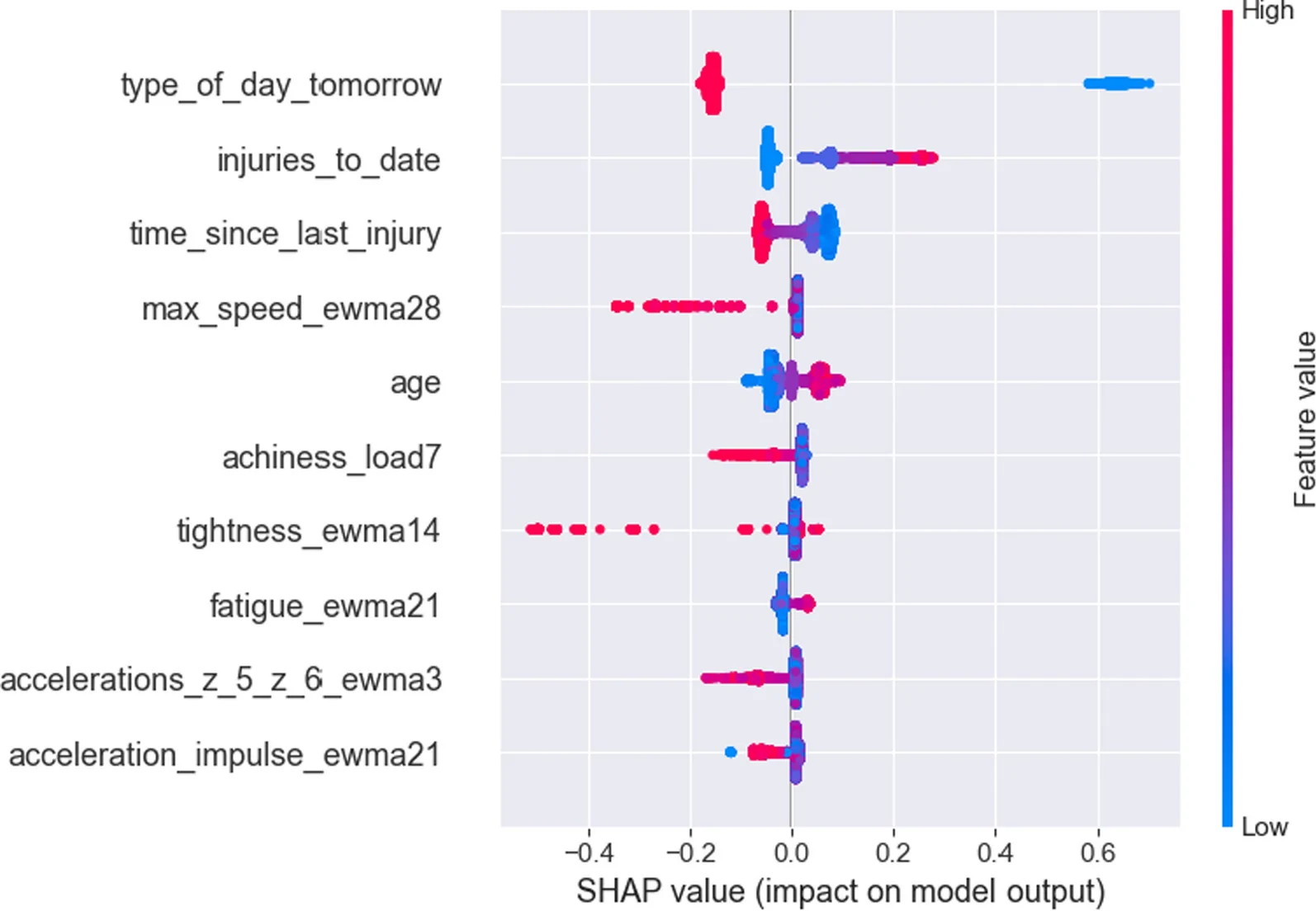
Summary plot of top 10 SHapley Additive exPlanations (SHAP) values for CatBoost model applied to testing data. *EWMA* Exponentially Weighted Moving Average

SHAP analysis, the most commonly utilised model explainability technique used in existing literature [22], was applied to quantify and visualise the marginal contribution of each feature to the model predictions, thereby identifying the variables with the greatest influence on the estimated injury risk. The global feature importance for the highest performing CatBoost model was computed using TreeExplainer, appropriate for tree-based models. This analysis indicated that *type_of_day_tomorrow* (i.e. whether the session is ‘training’ or ‘match’) had the greatest influence on the model’s outcome (Fig. 3). Moreover, the top three features each relate to non-modifiable risk factors—type of day tomorrow, injuries to date, time since last injury. The most influential GNSS workload metric was the 28-day exponentially weighted moving average of *max_speed* (i.e., the maximal speed achieved by a player in each session). Importantly, SHAP analysis should be interpreted as a measure of feature contribution to the model predictions, rather than evidence of causal relationships. Furthermore, the low predictive ability of the CatBoost model suggests that these estimates should be interpreted with caution.

## Discussion

Despite the diversity and granularity of the datasets available, the tested ML approaches demonstrated limited predictive ability within the modelling framework analysed in this study, primarily due to very low precision and high false-positive rates. Although the highest-performing model achieved a ROC AUC of 0.66—comparable with prior injury prediction literature, where 33% of studies reported ROC AUC values below 0.7 [22]—this level of discrimination did not translate into actionable decision support. For the purposes of the following discussion, all findings relate to the results from the highest performing model, CatBoost.

### Clinical Implications

From a practical standpoint, only eight of the 54 injuries (15%) incurred during the testing period were correctly predicted, accounting for a cumulative time-loss of 208 days (15%). The correctly predicted injuries related to six players, with two players each sustaining two correctly predicted injuries in the testing period.

Overall, during the 9-month testing period (13,833 observations) the model identified 626 instances (4.5%) in which a player was classified as being at an elevated risk of sustaining an injury in the subsequent match or training session. This equates to an average of four high-risk predictions per provincial team each week. In a practical context, this relates to 1 correct prediction for every 77 false predictions, demonstrating a very low positive predictive value. This high false-positive rate would likely lead to ‘alarm fatigue’, whereby excessive alerting would cause medical practitioners to become desensitised to warnings [48], thus limiting the model’s effective deployment within an elite sports environment.

Of the independent variables deemed most important by the SHAP analysis, four of the top five relate to non-modifiable risk factors. The most influential feature, *type_of_day_tomorrow*, reflects the increased injury incidence rate for matches (12.6 per 1000 h) compared to training sessions (2.4 per 1000 h) within the testing data. Moreover, history of previous injury (*injuries_to_date*) and *age* are both widely acknowledged risk factors for subsequent lower-limb injury [49].

The only GNSS measure within the top five SHAP values relates to the 28-day exponentially weighted moving average for *max_speed*, with the concentration of red dots on the left side of the plot indicating the protective effects of higher maximal speed reached per session (Fig. 3). This finding supports previously published research in which higher chronic sprint exposure has been shown to mitigate the risk of lower limb non-contact injury [50], although this must be caveated by the poor predictive performance of the model from which the SHAP values are derived.

**Fig. 4:**
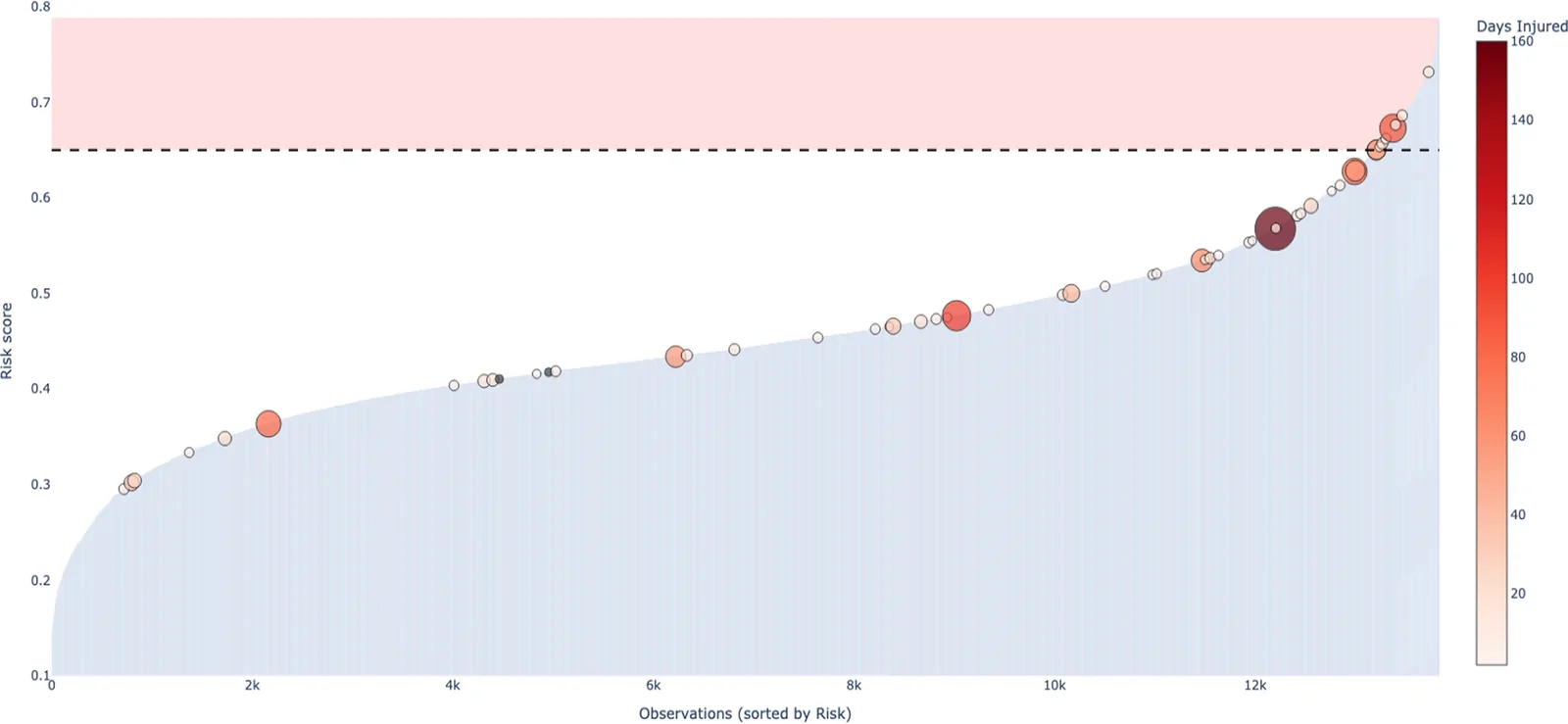
‘Injury risk’ score output from the Categorical Boosting (CatBoost) model applied to testing observations. Bubbles represent the 54 injury events during this period, with colour and size representing number of days injured (i.e. injury severity)

The risk scores generated from the CatBoost model for the testing dataset are visualised in Fig. 4. Annotated on this curve are the 54 injury events incurred during the testing period, with the colour and size of the points representing the severity of the injury (i.e. days injured). Injury events are concentrated at higher predicted risk levels, with 28 injuries (52%) occurring at risk scores ≥ 0.5, despite this region comprising 27% of all predictions. Conversely, the figure depicts the widely diffuse nature of the predicted risk scores, with injury events occurring across the entire risk profile. Injury severity is also not clearly correlated with increased predicted risk, demonstrating the models’ inability to discriminate between low and high burden injuries.

### Implications of Low Event Prevalence

By adopting a consensus-based definition of in-scope injury, this study sought to address the heterogeneity in injury definitions identified in previously published literature [22]. However, even with the grouping of different soft-tissue non-contact injuries which ignores specific muscle behaviour and injury risk, the chosen definition of injury led to a substantial reduction in event prevalence, resulting in severe class imbalance within the test set (prevalence = 0.39%). While event rarity alone does not explain the poor model performance, this extreme sparsity underscores the inherent difficulty of predicting sports injury events and limits the ability of ML models to reliably extract signal from such rare occurrences, constraining their capacity to predict future risk of injury. These findings suggest that the prediction of rare, appropriately defined sports injuries may lie beyond the practical limits of the tested supervised ML framework.

### Strengths and Limitations

To the best of the authors’ knowledge, the current study is the first to synthesise multi-season data from GNSS tracking devices, self-perceived wellness reporting, and musculoskeletal screening tests across multiple professional sports teams. The strength of this approach lies in the homogeneity of data sources, with identical hardware for GNSS, wellness, and musculoskeletal data collection in situ across the teams analysed. This standardisation extends to injury surveillance through the use of a common athlete management system, aiding consistent definition of the target variable.

The current study has several limitations to address. Firstly, although the novelty of this research lies within the multi-team approach, potential issues may be induced by including data from diverse club settings. These include the differences in playing styles of each team and the variations in playing surfaces used in training and match situations. Moreover, by selecting players with complete datasets across a 3-year period, selection bias may exist whereby athletes with greater availability are preferentially included. Injuries were not limited to the first occurrence per player, instead all events were incorporated in the analysis to reflect the recurrent nature of injuries in applied sports settings. This approach creates a repeated-measures structure within the data and therefore within-player correlation. Although individual injury history was partially accounted for through engineered features, these influences may limit the generalisability of the model. No thresholds were applied during the last observation carried forward method for filling missing musculoskeletal data, which may induce bias in the data if true physiological changes were not captured. Moreover, musculoskeletal data collection may be subject to non-uniform measurement frequency, given that players with clinical concerns, or those progressing through a data-informed return-to-play programme, are likely to be tested more frequently, which could introduce selection bias. Collinearity checks were not performed for engineered EWMA features, which may result in redundancy among independent variables, potentially impacting model interpretability. Predictive performance was evaluated using classification and discrimination metrics only, calibration and decision-analytic approaches were not assessed, which may limit the comprehensiveness of model evaluation. A sample size calculation was not conducted prior to analysis. As with all observational ML analyses, identified associations should not be interpreted causally.

## Conclusion

This study contributes to addressing key methodological limitations in sports injury risk prediction research, including imprecise injury definitions and reliance on single-team cohorts. However, predictive performance across the evaluated models was poor, with low precision scores highlighting each model’s inability to effectively identify impending injuries. The resulting high false-positive rates highlight the limited practical utility of the tested ML framework in real-world elite sport settings. Research may need to move beyond binary injury prediction toward models that support clinical reasoning, workload tolerance assessment, or individualised risk communication. Future work should seek to incorporate data captured during gym-based strength and conditioning sessions, or musculoskeletal testing conducted using electronic testing equipment with greater granularity, and ability to identify side-to-side asymmetries.

## Supplementary Information

Supplementary material 1.

Supplementary material 2.

## Data Availability

Due to governance and data protection regulations set by the IRFU and UCD research committees, the datasets used in this study cannot be made publicly available. These datasets contain sensitive athlete performance, health, and injury information collected within a professional sporting environment and are subject to contractual, confidentiality, and ethical restrictions designed to protect participant privacy and organisational interests.

## Abbreviations

AUPRC: Area under the precision-recall curve
AI: Artificial intelligence
CAP: Cumulative accuracy profile
CatBoost: Categorical boosting
EWMA: Exponentially weighted moving average
XGBoost: Extreme gradient boosting
GNSS: Global Navigation Satellite System
IOC: International Olympic Committee
IRFU: Irish Rugby Football Union
KNN: K-nearest neighbours
kg: Kilograms
MAR: Missing at random
ML: Machine learning
m: Metres
MICE: Multiple imputation by chained equations
OSIICS: Orchard Sports Injury and Illness Classification System
ROC AUC: Receiver operating characteristic area under the curve
SHAP: SHapley Additive exPlanations
SOAP: Subjective, objective, assessment, plan
SVM: Support vector machine
VIF: Variance inflation factor
