# Supplementary material for: Machine Learning Model Development and Evaluation for Non-contact Lower Limb Injury Risk Prediction in Elite Male Rugby Union: A Multi-team, Multi-season Analysis
Source: Sports Med Open. 2026 Aug 26;12:123. doi: 10.1186/s40798-026-01060-7 (PMC13518749; doi:10.1186/s40798-026-01060-7)
Supplement: Supplementary file 2 [file 40798_2026_1060_MOESM1_ESM.docx]

*Supplementary material for the manuscript:*

**Machine Learning Model Development and Evaluation for Non-Contact Lower Limb Injury Risk Prediction in Elite Male Rugby Union: A Multi-Team, Multi-Season Analysis**

Journal

Sports Medicine – Open

Authors

Chris Leckey^1,2^, Nicol van Dyk^1,3^, Jakim Berndsen^1^, Cailbhe Doherty^1,4^, Aonghus Lawlor^4^, Eamonn Delahunt^1,5^

^1^ School of Public Health, Physiotherapy & Sports Science, University College Dublin, Dublin, Ireland.
^2^ High Performance Unit, Irish Rugby Football Union, Dublin, Ireland.
^3^ Section Sports Medicine, Faculty of Health Sciences, University of Pretoria, Pretoria, South Africa.
^4^ Insight Centre for Data Analytics, University College Dublin, Dublin, Ireland
^5^ Institute for Sport and Health, University College Dublin, Dublin, Ireland.

Corresponding Author

Chris Leckey, High Performance Unit, Irish Rugby Football Union, Dublin, Ireland
E-mail address: [christopher.leckey@ucdconnect.ie](mailto:christopher.leckey@irfu.ie)

**SUPPLEMENTARY MATERIALS**

| **Supplementary Table S1:** List of independent variables available for analysis following data pre-processing. | | | |
| --- | --- | --- | --- |
| **Dataset** | **Feature** | **Description** | **Feature Name** |
| GPS Workload^†^ | Acceleration Impulse | Acceleration intensity per minute calculated using cumulative magnitude divided by number of occurrences. | *acceleration_impulse* |
|  | Accelerations | Accelerations between 4.0-10 m/s/s with minimum duration of 0.5s. | *accelerations_z_5_z_6* |
|  | (Zone 5 to Zone 6) |  |  |
|  | Average Metabolic Power | Average power output in Watts per kg bodyweight per second during drill/session. | *average_metabolic_power* |
|  | Collision Load | Calculation of stress and toughness of collisions based on magnitude and duration of each collision. | *collision_load* |
|  | Decelerations | Decelerations between 4.0-10 m/s/s with minimum duration of 0.5s. | *decelerations_z_5_z_6* |
|  | (Zone 5 to Zone 6) |  |  |
|  | Distance Covered | Distance covered in relative speed zone 6. | *distance_z_6_relative* |
|  | (Zone 6 Relative) |  |  |
|  | Dynamic Stress Load | Weighted total of accelerometry above 2G based on convex curved G-force ratings. | *dynamic_stress_load* |
|  | Fatigue Index | Speed Intensity score divided by Dynamic Stress Load. | *fatigue_index* |
|  | High Metabolic Load Distance | Distance covered accelerating/decelerating above 2m/s² magnitude or above 5.5m/s. | *hml_distance* |
|  | High-Speed Running Per Minute (Relative) | Distance covered travelling above relative Zone 5 threshold per minute. | *hsr_per_minute_relative* |
|  | Lower Speed Loading | All accelerometer loading recorded while travelling at speeds lower than 2m/s. | *lower_speed_loading* |
|  | Max Speed | Maximum speed attained per day. | *max_speed* |
|  | Sprint Distance | Distance covered at a speed of ≥ 5.5 m/s (minimum duration of a sprint is 1 second). | *sprint_distance* |
|  | Total Time | Total duration (minutes) of training sessions and/or matches completed. | *total_time_minutes* |
| Self-Perceived Wellness^†^ | Achiness | Likert scale (0-10) response to: "Please rate your overall level of muscle soreness (achiness) today." | *achiness* |
|  | Appetite | Likert scale (0-10) response to: "Have you been 'off your food' in the past 24 hours?" | *food* |
|  | Fatigue | Likert scale (0-10) response to: "Please rate your overall fatigue (tiredness) levels today." | *fatigue* |
|  | Sleep* | Likert scale (0-10) response to: "Please rate your overall sleep quality (restfulness) from last night." | *sleep* |
|  | Stress | Likert scale (0-10) response to: "Please rate your overall stress levels today." | *stress* |
|  | Tightness | Likert scale (0-10) response to: "Please rate your overall level of muscle and/or joint stiffness (tightness) today." | *tightness* |
| Musculoskeletal Testing^†^ | Groin Squeeze | Measured using a sphygmomanometer at 45° hip flexion.^17^ | *groin_squeeze* |
|  | Ankle Dorsiflexion | Measured during a “Lunge”/”Knee to Wall” test.^18^ | *ankle_dorsiflexion* |
|  | Sit & Reach | Measured during a standard “Sit & Reach” test.^19^ | *sit_reach* |
| Non-Modifiable Risk Factors | Height | Player weight (kg) | *height* |
|  | Weight | Player height (cm) | *weight* |
|  | Age | Player age (anonymised to age at first day of each season). | *age* |
|  | Position | Playing position grouped as 'Front Row', 'Second Row', 'Back Row', 'Half Backs', 'Centres', 'Back Three'. | *position_new* |
|  | Historic Injury Profile (All time, all time-loss injuries) | Rolling total of all injuries sustained prior to the commencement of the study. | *ll_inj_hist* |
|  | NC-TL-LLST injuries sustained in the study period | Rolling total of non-contact, lower limb, soft tissue injuries sustained during the study period. | *injuries_to_date* |
|  | Days since last NC-TL-LLST injury | Days since the last in-scope injury. | *time_since_last_injury* |
|  | Medical illnesses reported in last 30 days | Number of illnesses logged in the previous 30 days. | *illnesses_in_last_month* |
|  | Non time-loss injuries reported in last 30 days | Number of logged interactions with medical team that resulted in no time-loss in the previous 30 days. | *ntl_injs_in_last_month* |
|  | Concussions sustained in study period | Rolling total of recorded concussions sustained during the study period. | *concussions_in_study* |
|  | Match minutes played | Competitive minutes played provincially/internationally. | *match_time_total* |
|  | Type of day (Tomorrow) | Will the next day be 'Off Feet Day', 'Training Day', or 'Match Day'. | *type_of_day_tomorrow* |
|  | Week of season | Week number to denote phase of the season. | *week_of_season* |
| * Sleep score subtracted from 10 to reflect sentiment of other Likert scale data.  † Raw daily features dropped in place of temporal representations (3-,7-,14-,21-28-day EWMA, week-to-week % change, 7-day cumulative load)  NC-TL-LLST = Non-contact, time-loss, lower limb, soft tissue injury | | | |

**STATISTICAL ANALYSES**

**Receiver operating characteristic area under the curve (ROC AUC)** *=* Measures a binary classifier’s ability to distinguish between classes across all thresholds, with values ranging from 0.5 (random guess) to 1 (perfect discrimination).

**Threshold** = The cut-off point of risk score above which model predictions were classified as ‘1’, optimised by F1-Score.

**Precision** is the fraction of positive cases correctly identified among all cases identified.

$$Precision=\frac{TP}{TP+FP}$$

**Recall**, or sensitivity, is the fraction of positive cases correctly identified among all positive cases.

$$Recall =\frac{TP}{TP+FN}$$

where TP = True Positives, FP = False Positives, TN= True Negatives, FN = False Negatives

**F1-Score** is the harmonic mean of precision and recall, where 1 indicates perfect precision and recall, and 0 represents the lowest possible value.

$$F1\cdot Score=\frac{2 \times Precision \times Recall}{Precision \times Recall}$$

**Prevalence** is the proportion of the cohort who sustained an injury in the study period.

$$Prevalence=\frac{Injuries}{Injuries+Non\cdot Injuries}$$

**Area under the precision-recall curve (AUPRC)** = Measures a binary classifier’s ability to distinguish between classes across all thresholds, with values ranging a maximum of 1 (perfect discrimination) to a lowest value equal to the prevalence of the positive class.

**CAP CURVE FOR CATBOOST**

**OPTIMISED HYPERPARAMETERS**

*Logistic Regression*

F1-Score optimised threshold = 0.5

| *C* | 0.129 |
| --- | --- |

*Support Vector Machine*

F1-Score optimised threshold = 0.01

| C | 0.003 |
| --- | --- |
| gamma | 0.0003 |

*Random Forest*

*F1-Score optimised threshold =* 0.01

| *n_estimators* | 338 |
| --- | --- |
| *max_depth* | 10 |
| *min_samples_split* | 8 |
| *min_samples_leaf* | 3 |

*XGBoost*

F1-Score optimised threshold = 0.52

| *n_estimators* | 267 |
| --- | --- |
| *max_depth* | 4 |
| *learning_rate* | 0.007 |
| *subsample* | 0.775 |
| *colsample_bytree* | 0.757 |
| *reg_lambda* | 6.121 |
| *reg_alpha* | 3.950 |

*CatBoost*

F1-Score optimised threshold = 0.65

| *iterations* | 300 |
| --- | --- |
| *depth* | 2 |
| *learning_rate* | 0.020 |
| *l2_leaf_reg* | 8.266 |
| *subsample* | 0.933 |
| *loss_function* | LogLoss |
